# From Acid Activation Mechanisms of Proton Conduction to Design of Inhibitors of the M2 Proton Channel of Influenza A Virus

**DOI:** 10.3389/fmolb.2021.796229

**Published:** 2022-01-14

**Authors:** Elnaz Aledavood, Beatrice Selmi, Carolina Estarellas, Matteo Masetti, F. Javier Luque

**Affiliations:** ^1^ Departament de Nutrició, Ciències de l'Alimentació i Gastronomia, Institut de Biomedicina and Institut de Química Teòrica i Computacional, University of Barcelona, Barcelona, Spain; ^2^ Department of Pharmacy and Biotechnology, Alma Mater Studiorum – Università di Bologna, Bologna, Italy

**Keywords:** M2 proton channel, influenza A virus, proton transport, inhibition, molecular mechanism, drug design

## Abstract

With an estimated 1 billion people affected across the globe, influenza is one of the most serious health concerns worldwide. Therapeutic treatments have encompassed a number of key functional viral proteins, mainly focused on the M2 proton channel and neuraminidase. This review highlights the efforts spent in targeting the M2 proton channel, which mediates the proton transport toward the interior of the viral particle as a preliminary step leading to the release of the fusion peptide in hemagglutinin and the fusion of the viral and endosomal membranes. Besides the structural and mechanistic aspects of the M2 proton channel, attention is paid to the challenges posed by the development of efficient small molecule inhibitors and the evolution toward novel ligands and scaffolds motivated by the emergence of resistant strains.

## Introduction

Influenza A virus is an important pathogen that still causes the death of 290,000–650,000 people in seasonal outbreaks worldwide (Iuliano et al., 2018). It pertains to the Orthomyxoviridae family, which is characterized by the presence of a negative-sense, single-stranded, enveloped ribonucleic acid with a segmented genome (Cheung and Poon, 2007). There are four genera in the Orthomyxoviridae family: Influenza A, Influenza B, Influenza C, and Thogotovirus. Influenza A viruses are found in many different animals, and Influenza B viruses circulate widely only among humans. Influenza A viruses are divided into subtypes based on two proteins on the surface of the virus: hemagglutinin (HA), which is responsible for the receptor binding and membrane fusion, and neuraminidase (NA), which assists the release of the viral progeny (Cheung and Poon, 2007). Up to 18 variants of HA (from type H1 to H18) have been described till now, whereas 11 subtypes have been disclosed for NA. All known subtypes of influenza A viruses have been found among birds, except subtypes H17N10 and H18N11, which have only been found in bats. Only some subtypes (H1N1, H2N2, H3N2, H5N1, H7N7, and H9N2) have been isolated from humans, suggesting that there are restrictions to host viruses in humans.

While infections in humans directly from animals are unusual, sporadic infections and outbreaks caused by influenza A viruses have occurred in the past. The most severe pandemic was the “Spanish Flu” of 1918–1919, which caused 20–50 million deaths by influenza A H1N1 virus strain. The subsequent health challenges were the “Asian flu” in 1957 and the “Hong Kong flu” in 1968, which were caused respectively by H2N2 and H3N2 strains, and more recently the 2009 swine influenza pandemics, which pertains to the H1N1 strain (Cox and Subbarao, 2000; Hayward et al., 2014; Watson et al., 2015; Saunders-Hastings and Krewski, 2016). The latent risk of facing future pandemics underlined the Global Influenza Strategy initiative launched by the World Health Organization in 2019, which has the goal of preventing seasonal influenza, controlling the spread of influenza from animals to humans, and preparing for the next influenza pandemic (World Health Organization, 2019). This threatening scenario underscores the urgency in developing better diagnostic tools and promoting the discovery of effective influenza treatments, including vaccines and antiviral drugs.

This review aims to discuss experimental and computational efforts invested in elucidating the structural basis of the proton conducting activity of the M2 protein channel and the discovery of antiviral compounds targeting the M2 transmembrane domain. To this end, the manuscript first describes the role of the M2 proton channel in the replicative life cycle of the influenza A virus. Then, attention is focused on the structure of the proton channel, which is directly implicated in the proton transfer across the viral membrane as a preliminary event leading to the fusion of viral and host membranes and the subsequent release of the viral genetic material. Emphasis is made on the molecular mechanisms that underlie the proton conduction through the lumen of the M2 proton channel. The discussion also highlights the role played by key residues located in the transmembrane region, and especially the efforts spent in the determination of the pKa of the imidazole rings present in the His37 tetrad. Finally, the manuscript examines the mechanism of action of selected inhibitors, and the evolution followed in the design of novel compounds introduced to increase the inhibition potency and to mitigate the emergence of resistance to current treatments.

## The Influenza A Virus Replicative Cycle

Influenza A virus replicates in the epithelial cells of the upper respiratory tract of humans, pigs, and horses. Regarding *in vitro* models, the virus can also affect different cells that contain targets decorated with sialic acid moieties (Julkunen et al., 2001; Sidorenko and Reichl, 2004), which act as the entrance anchor point into the cells.

Enclosed by a lipid-protein envelope, the genetic material of the influenza A virus contains 8 single-stranded RNA segments that encode the genetic information for viral proteins (Dou et al., 2018; Krammer et al., 2018; Jung and Lee, 2020): the membrane-bound hemagglutinin (HA) and neuraminidase (NA), the transmembrane M2 proton channel, the matrix protein M1, the non-structural proteins (NS1, NS2/NEP), and the heterotrimeric RNA-dependent RNA polymerase (RdRp), formed by three domains (PA, PB1, and PB2), and nucleoprotein (NP) components.

**FIGURE 1:**
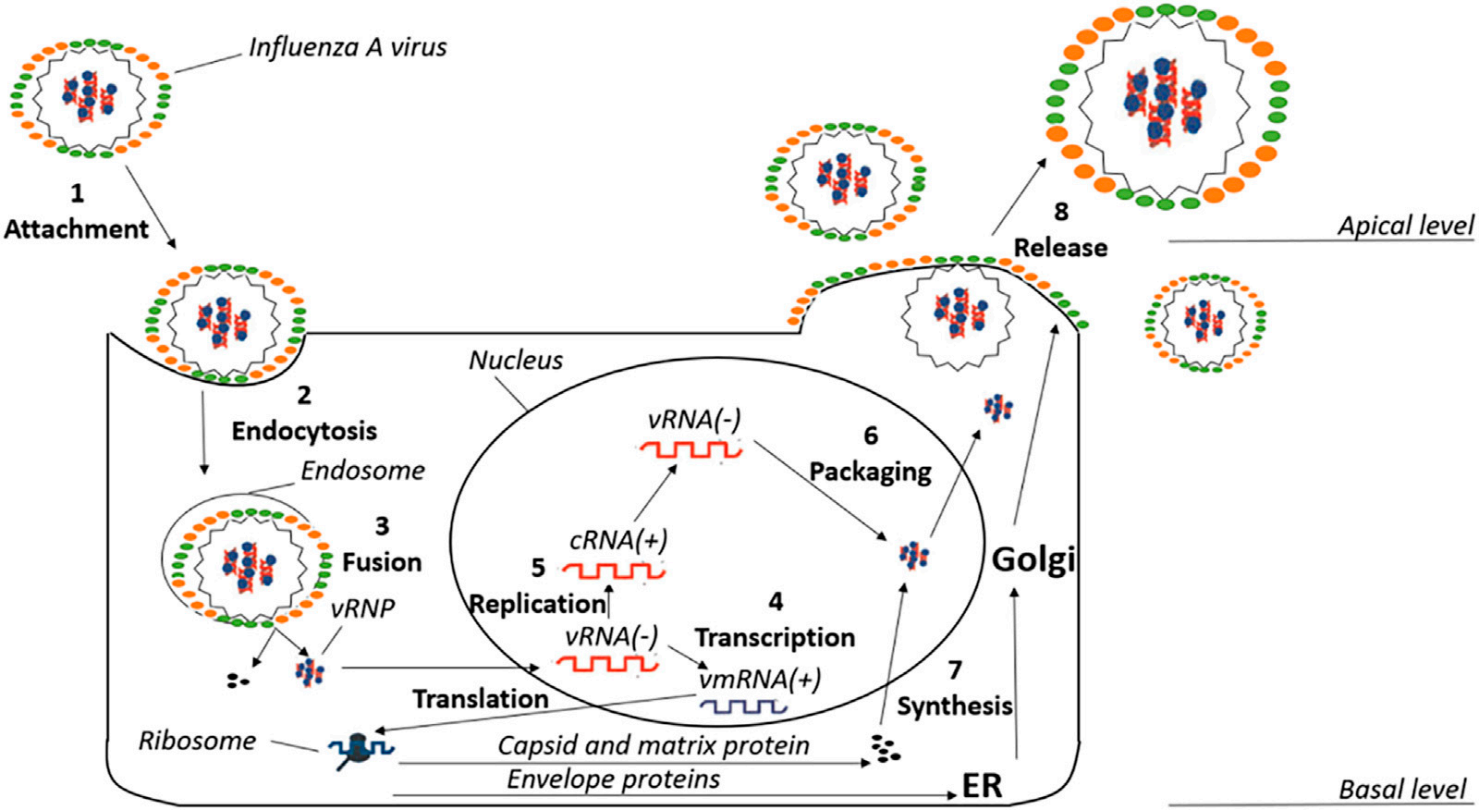
Schematic pattern of the different phases of the replicative cycle in influenza A virus.

The replicative life cycle of the virus is schematically shown in Figure 1. The first step of the cycle is the entry of the virus inside the host cell (step 1). Viral entry into the cell occurs via HA, which recognizes and binds the sialic acid of the host cell receptors. The virions penetrate inside the host cell through clathrin-dependent receptor-mediated endocytosis (step 2). The acidification of the endosome is facilitated by the M2 proton channel, which transfers protons to the interior of the viral particles. This event promotes a large-scale conformational remodeling in HA that facilitates the release of the fusion peptide, and the concomitant structural rearrangement of HA triggers the fusion of the viral and host membranes, leading to conformational changes in the M1 protein that facilitate the release of the viral ribonucleoproteins (vRNPs) to the cellular cytoplasm (step 3) (Honda et al., 2002). These vRNPs comprise complexes formed by the individual viral RNA segments and proteins, including the RNA-dependent RNA polymerase (RdRp), which is associated with the 3′- and 5′-ends of viral RNA segments, and nucleoproteins (NPs) that are coating the remainder of the viral RNA. The vRNP complexes are then transported into the nucleus to initiate transcription and replication by RdRp, which is formed by the polymerase basic protein 1 (PB1), polymerase basic protein 2 (PB2), and polymerase acidic protein (PA). The first step is known as cap-snatching, a process where RdRp (PB2 and PA subunits) interacts with the C-terminal domain of the host RNA polymerase II and removes the first 10–13 nucleotides of a nascent transcript, which is then used as a primer to initiate transcription from the viral RNA template by PB1 (De Vlugt et al., 2018). This is followed by a viral genome replication phase that involves the synthesis of full-length viral RNA and cRNA strands (steps 4 and 5) (Pielak and Chou, 2011). With a late stage of packaging, vRNPs are formed (step 6). The synthesis of M2 protein, HA and NA is carried out by ribosomes bound to the membranes of the endoplasmic reticulum, which are then glycosylated and transported to the Golgi apparatus (step 7) (Sidorenko and Reichl, 2004). Finally, a complex between the membrane-embedded proteins and vRNPs is formed and delivered to the cell membrane, leading to the assembly of new virions that will be released to the extracellular side of the host cell membrane (step 8).

The preceding discussion suffices to reveal the complexity encoded in the multi-step processes - cell entry, replication, intracellular trafficking, virion assembly, and release - involved in the generation of new virions. In this scenario, the search for effective antiviral treatments has been inspired by the identification of druggable viral targets with a relevant role in the life cycle of virus infection. Early drug discovery projects targeting the M2 proton channel primarily relied on adamantane-related compounds (Moorthy et al., 2014; Wang et al., 2015). However, the emergence of resistant strains motivated the search of novel therapeutic approaches, including the development of 1) NA inhibitors such as zanamivir and oseltamivir, although drug-resistant variants have also been reported, 2) HA inhibitors, as exemplified by the discovery of arbidol and JNJ7918, and, more recently, 3) RdRp inhibitors, such as pimodivir and baloxavir (Loregian et al., 2014; Wu et al., 2017; Han et al., 2018; Mifsud et al., 2019; Ginex and Luque, 2021). The discovery of small-molecule antiviral compounds against these targets has benefited from the progress made in disclosing the fine structural details of these proteins, the molecular mechanisms that underlie their biological function, and the characterization of the mode of action of inhibitory compounds.

## Structure of the M2 Proton Channel

The M2 proton channel is a homotetrameric integral transmembrane (TM) protein located in the viral envelope of the influenza A virus. It is a selective, pH-dependent channel that regulates the acidification of the interior of the virion, leading to dissociation of the viral RNA from its bound matrix proteins and release of the viral genetic material for replication (Pinto et al., 1992; Pielak and Chou, 2011; Manzoor et al., 2017).

**FIGURE 2:**
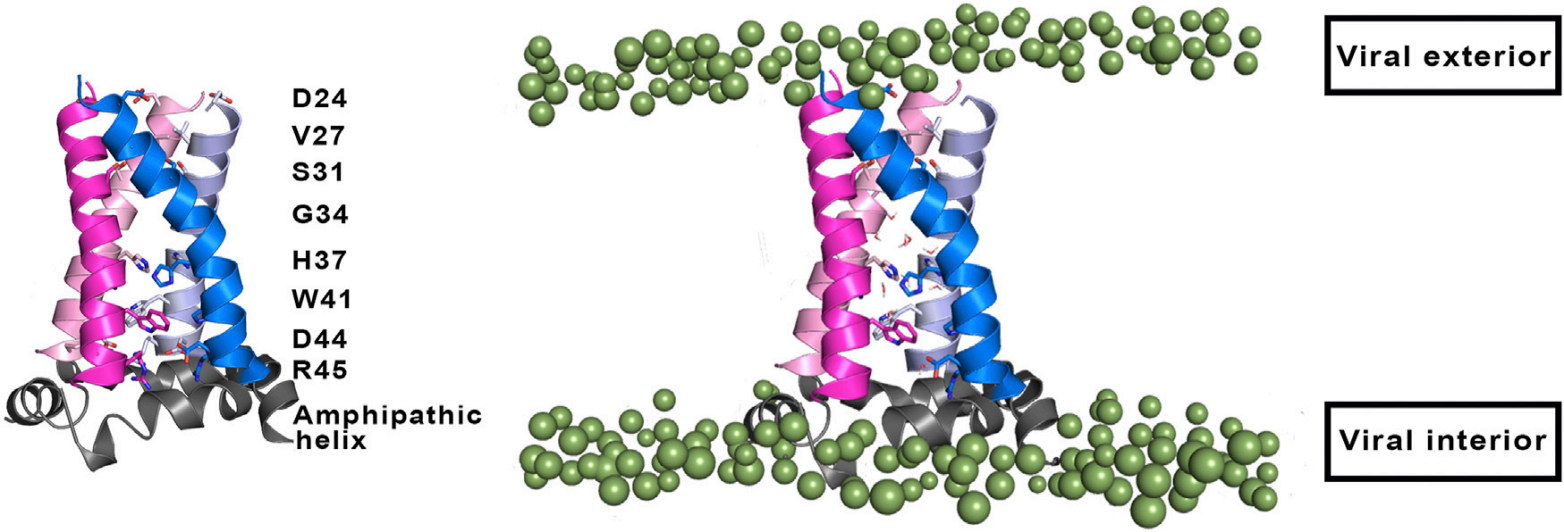
(Left) Representation of selected residues that shape the inner pore of the M2 proton channel (shown as sticks). (Right) Representation of the four transmembrane helices (residues 22–46; shown as cartoon in magenta, blue, pink and violet), where the amphipathic helices (residues 47–67) that are located near the inner side of the membrane are shown as grey cartoon. Polar heads of membrane lipids in both outer and inner sides of the viral membrane are shown as green spheres (adapted from a Molecular Dynamics simulations of the membrane-embedded M2 proton channel).

From a structural point of view, the M2 channel is a homotetramer, each monomer consisting of 97 amino acids and can be divided into four main regions: 1) the highly conserved, unstructured N-terminal domain (residues 1–21) located in the viral exterior, which assists the incorporation of the M2 channel into the virion, 2) the TM domain (residues 22–46) (Figure 2), which assembles into a tetrameric domain and is involved in the proton flux, 3) an amphiphilic membrane-anchored helical domain (residues 47–67), which is located in the interface with the membrane and stabilizes the channel by inducing membrane curvature and mediates membrane scission, and 4) the C-terminal domain (residues 68–97), which is located in the viral interior and interacts with the matrix protein M1 and contributes to virus packaging and budding (McCown and Pekosz, 2006; Rossman et al., 2010; Wang and Hong, 2015a).

The TM region contains the binding site for M2 inhibitors in the interior of the pore, filling a pocket located between the tetrads formed by residues Val27, Ser31, Gly34, and His37 (Schnell and Chou, 2008; Acharya et al., 2010; Cady et al., 2010; Sharma et al., 2010). This pore is implicated in the proton conduction, and the proton current is regulated by the tetrads formed by His37 and Trp41. His37 is a pH sensor and can conduct the proton inside the channel by changing the protonation state of the imidazole side chain, whereas Trp41 acts as a gate of the channel. Accordingly, binding of compounds that fill the binding site in the interior of the M2 channel would sterically impede the passage of protons (Figure 2).

The pH-sensing gating mechanism regulates the proton flux through the channel, which is modulated by the transition from an open state at an acidic pH, where the imidazole rings of the His37 tetrad are protonated, to a closed state at alkaline pH environment, which promotes the deprotonated state of His37 residues. In the open state, the M2 channel is also permeable to some cations, such as Na^+^ and K^+^, through an antiporter-like mechanism, carrying out the metal cation efflux in conjunction to proton influx, but the permeability to protons has been estimated to be 10^6^–10^7^ higher than the conduction of alkaline cations (Chizhmakov et al., 1996; Shimbo et al., 1996; Mould et al., 2000a; Manzoor et al., 2017). There is an asymmetric conductance as the proton flux is higher when the pH outside the virus is low, consistently with an acidic endosome. This behavior has been attributed to the presence of the Trp41 gate, which reduces the conduction in the reverse direction (Ma et al., 2013). Thus, when the His37 tetrad is protonated by protons coming from the endosome, the indole moiety of Trp41 undergoes a conformational change and the channel enters an open state that would allow the passage of protons to the interior of the virus.

The functional relevance of His37 and Trp41 is revealed by the drastic effects triggered by mutations in these positions, which cause a reduction in the pH sensitivity of the channel, and a disruption in the proton conduction (Wang et al., 1995; Tang et al., 2002; Venkataraman et al., 2005). Thus, mutations in His37 have a drastic effect on the functional properties of the M2 channel, since the mutated proteins not only increase the proton conduction of the channel, but also suppress the selectivity for proton conduction. As an example, the specific activity determined for the voltage-independent component of the conductance at pH 6.2 for the wild type channel was 0.16 μA/ng, but increased to 1.36 and 30 μA/ng for the H37G and H37E mutated channels, respectively (Holsinger et al., 1994). Furthermore, the mutated ion channels H37G, H37S, and H37T were found to be deficient in ion selectivity (Venkataraman et al., 2005). On the other hand, the expression of the M2 protein in oocytes leads to an inward H^+^ current upon bathing in acidic media (pH 5.9), which is lost upon return to alkaline conditions (pH 8.5). However, whereas no currents were observed for mutated channels containing Phe41, Cys41, or Ala41 prior to acidification, outward H^+^ currents were detected by changing acidic conditions to alkaline solution.

### Mechanism of Proton Conduction

The specific role played by His37 and Trp41 in assisting the proton flux through the pore of the M2 channel has been the subject of intense efforts, mainly focused on the TM domain, which reproduces most of the biophysical, site-directed mutagenesis and electrophysiological features of the full-length protein. These studies, further assisted by X-ray crystallography of structures solved in membrane-mimetic solvents at different pH and temperature, solution NMR, solid-state NMR (ss-NMR) spectroscopy (see below), in conjunction with computational simulations, have crystallized in several models of proton conduction.

**FIGURE 3:**
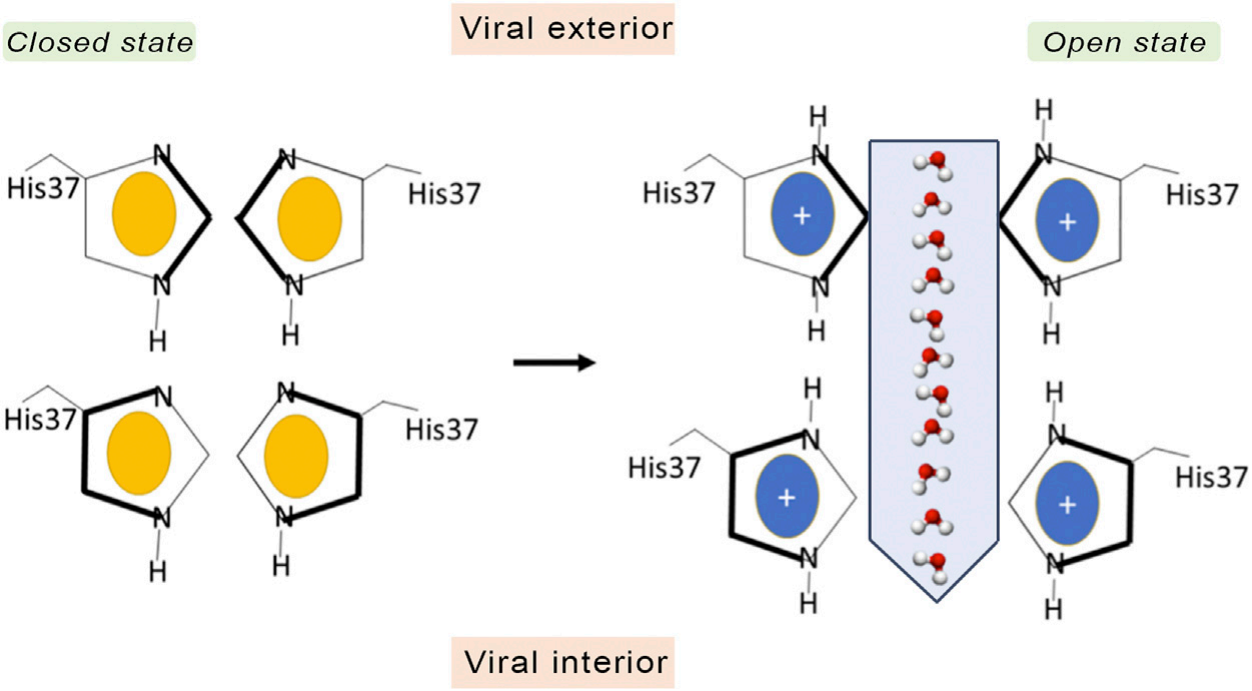
Schematic representation of the shutter mechanism. In the closed state the channel is in the neutral state (imidazole ring in yellow), and there is not an electrostatic repulsion between the imidazole rings of His37. However, when His37 are fully protonated (coloured in blue), the electrostatic repulsion between imidazole rings triggers a conformational change that allows water and protons to pass through the gate.

An early mechanistic hypothesis was the water wire model, also known as the shutter mechanism (Figure 3), which assumes the presence of a discontinuous water column when the His37 tetrad is in the deprotonated state, where the channel adopts a closed conformation that would be populated at alkaline pH (Sansom et al., 1997). When the pH decreases, protonation of the His37 tetrad leads to electrostatic repulsion between the charged imidazole rings. This would facilitate the conformational transition to an open state, which enables the diffusion of excess proton through the pore by means of a Grotthuss-type mechanism (Agmon, 1995). By merely working as a gatekeeper, in this model, the His37 tetrad would play a passive role as long as it is not directly involved in the proton transfer.

**FIGURE 4:**
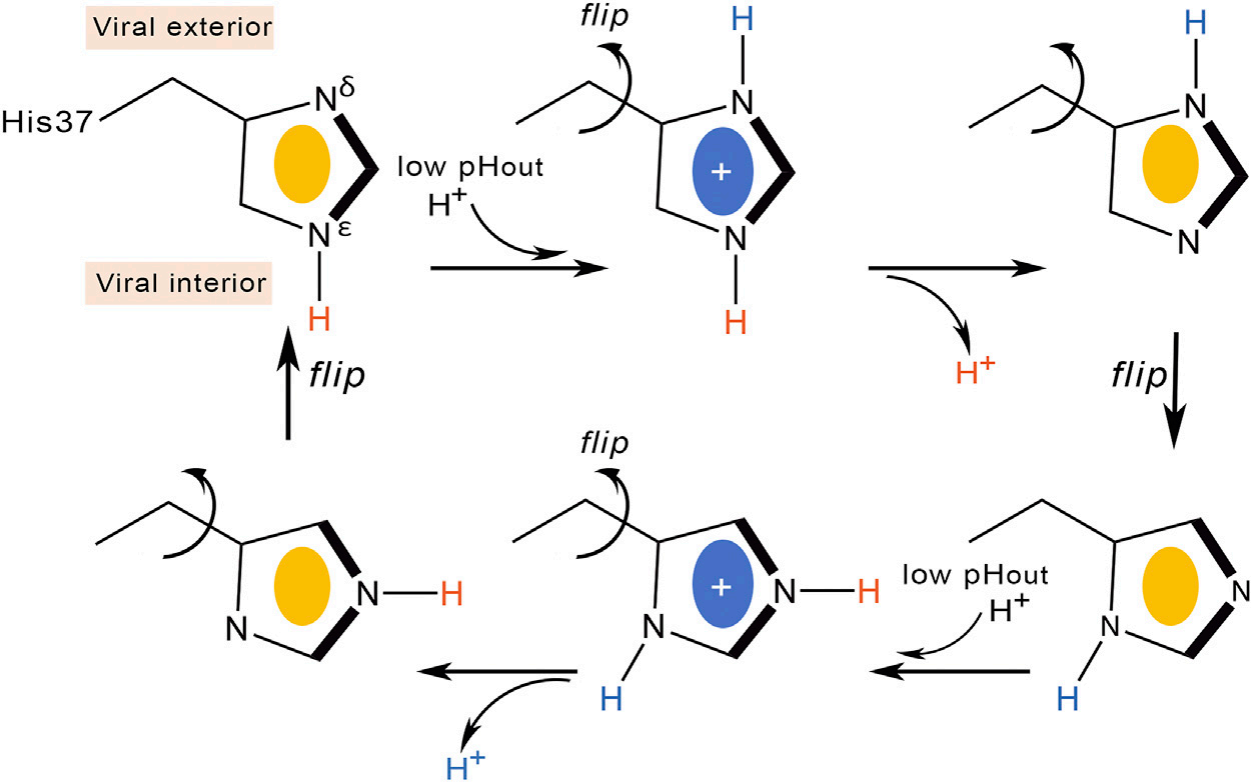
Schematic representation of the proton transfer flow associated to conformational rearrangement of the His37 imidazole ring.

In contrast to the shutter model, the currently accepted proton conduction mechanism assumes a proton relay model (Figure 4). According to this model, protons diffuse along a water wire until reaching the tetrad formed by His37, generating an imidazolium intermediate, which in turn releases a proton to the inner side (Pinto et al., 1997; Okada et al., 2001). This mechanism was first proposed by Pinto et al. on the basis of conductance measurements in conjunction with Cys scanning mutagenesis (Pinto et al., 1997). However, a number of questions remained to be elucidated, such as the number of His residues required to be protonated for proton conduction, the stabilization of the protonated imidazolium via interactions with other residues in the channel or with water molecules filling the inner pore, and whether translocation of the proton into the viral interior would be mediated by prototropic tautomerism of the imidazole ring or through conformational flip of the His37 side chain.

Deeper knowledge into the molecular events implicated in proton conduction has been gained from ss-NMR studies. Hu et al. refined the proton relay model suggesting that proton conduction is concomitant with His37 conformational changes (Figure 4) (Hu et al., 2010). At high external pH the His37 imidazole are tightly packed through CH-π interactions, preventing the formation of hydrogen-bonded water chains in the pore. Acidification protonates the imidazole ring at position Nδ1, and the electrostatic repulsion between protonated histidines would widen the pore (Khurana et al., 2009), enabling the reorientation of the imidazolium ring at the microsecond time scale. Proton conduction would involve imidazolium deprotonation facilitated by Cε1-Hε1 hydrogen bonding and continuous ring flips, which would contribute to the proper alignment of the charged imidazolium with C-terminal water molecules and rearrangement of the neutral imidazole to the N-terminal side of the pore to start another protonation cycle.

This proton transfer model dynamically couples the interaction of His37 with water molecules, which are required for delivering protons to the imidazole ring, with the conformational flips of His37 side chains. The barrier of the conformational rearrangement of the His37 residues was estimated to be close to 14 kcal mol^−1^, which would justify the temperature dependence of proton transport, though possibly this value may contain a contribution arising from the barrier for proton transfer (Lin and Schroeder, 2001). This model is consistent with the deuterium isotope effects (Mould et al., 2000b), and support comes from ^15^N NMR data, which yielded an estimated rate of 10^5^ s^−1^ for the protonation and deprotonation of the imidazole nitrogens (Hu et al., 2012). This rate was consistent with the ring reorientation rate estimated from motionally averaged dipolar couplings, supporting the proposal that ring reorientation is synchronized with, and facilitates, proton transfer. Notably, only a single histidine residue is required to be actively involved in proton transfer according to this mechanism.

**FIGURE 5:**
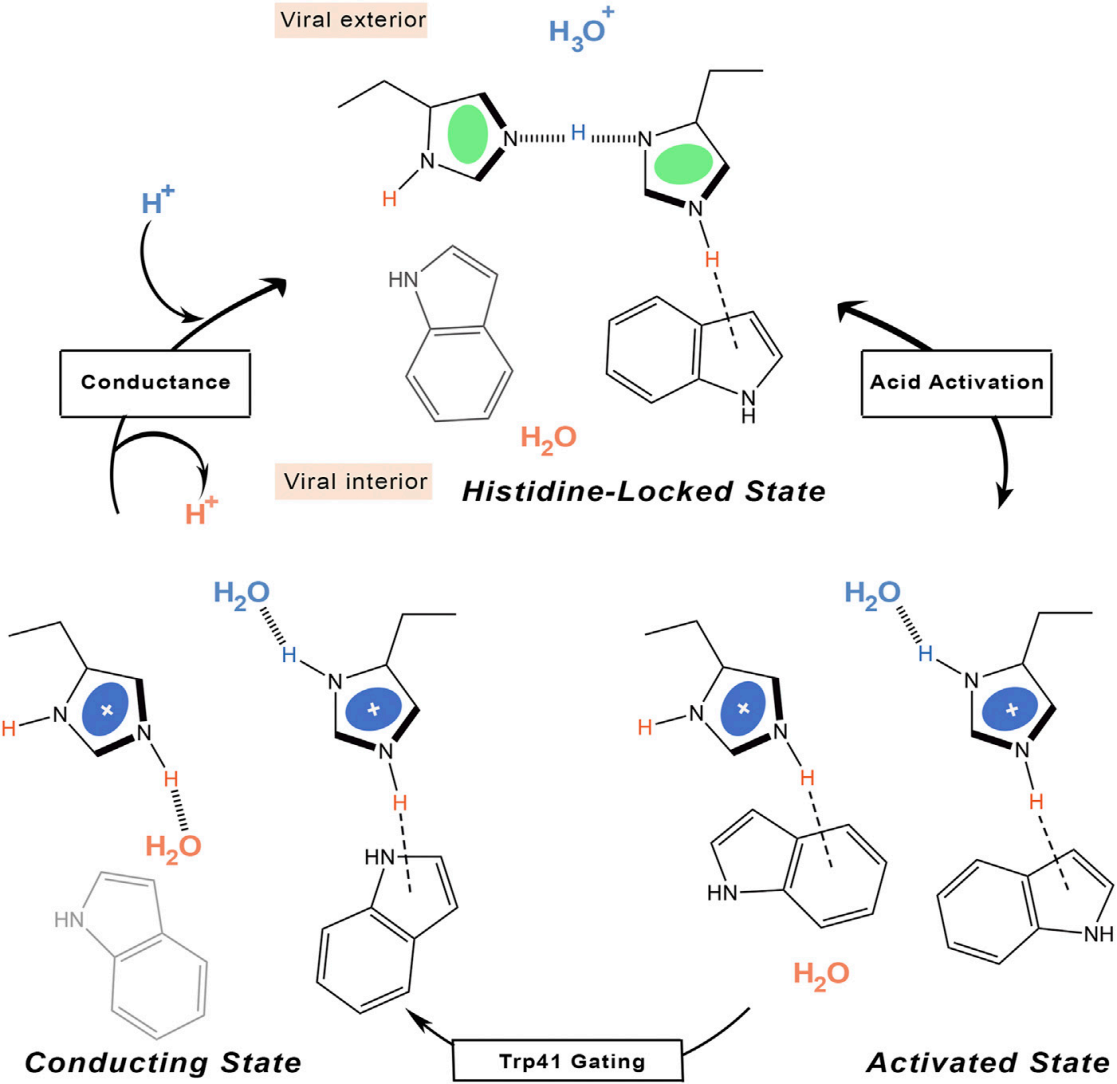
Schematic representation of the proton transfer flow associated to formation of hydrogen-bonded interactions between His37 residues.

Other ssNMR studies suggested that proton conduction may also involve the formation and breaking of hydrogen bonds between adjacent pairs of histidines, in conjunction with the assistance of tryptophan residues, to guide the proton through the channel (Figure 5) (Sharma et al., 2010). Thus, two His37 residues are protonated, each proton being shared with an adjacent His residue through a strong hydrogen bond. Accordingly, the His37 tetrad can be viewed as a pair of imidazole-imidazolium dimer (also denoted dimer-of-dimers). This would define the so-called histidine-locked state, which is further stabilized by a cation-π interaction of the protonated histidine with a tryptophan residue. When the pH decreases, a proton is transferred from the hydronium cation to the interresidue hydrogen bond formed between Nδ1 and Nε2 of the hydrogen-bonded histidines, leading to the activated state that contains a third protonated His37. The two imidazolium rings can then rotate, so that a protonated histidine may form a hydrogen bond with water in the N-terminal pore, while a conformational change in the other histidine enables the formation of a cation-π interaction with an indole of Trp41, which would block water access from the C-terminal pore. The conducting state is obtained when this indole moves aside to expose the Nε2 proton to a water molecule in the C-terminal pore. Finally, the histidine-locked state is recovered after the release of the Nε2 proton to a water molecule in the C-terminus. Recent studies have also suggested that the hydrogen bonding between imidazole rings could be formed even at a pH of 7.8 in the neutral charge state (Movellan et al., 2020), and that a water molecule is hydrogen-bonded to the deprotonated nitrogen of the histidine imidazole (Movellan et al., 2021).

According to this mechanism, the His37 tetrad would only sense acidification in the N-terminal side, reflecting previous observations obtained upon application of a voltage to drive protons outward in M2-transformed cells (Chizhmakov et al., 2003). The selectivity of proton selective transport is also justified as long as the proton must bind to and unbind from the histidine imidazole ring, which would explain the saturation attained at a moderate pH close to the histidine-tetrad pKa on the N-terminal side (Yi et al., 2009). In fact, the proton flux determined for the H37A mutant exhibits no pH dependence (Venkataraman et al., 2005). Finally, the low conductance would reflect the conformational transition of the Trp41 gate, which opens occasionally to form the conducting state that enables the proton to be released to the C-terminal pore.

More recently, measurement of ^15^N–^15^N J-couplings of ^15^N His37-labeled full-length M2 proton channel have been used to examine the occurrence of various imidazolium-imidazole hydrogen-bonding arrangements in the channel at low pH (Miao et al., 2015; Fu et al., 2020). According to these results, the proton conduction takes place through a dynamical process where low-barrier hydrogen bonds between pairs of His37 residues are broken and reformed.

From a computational point of view, the translocation of the proton has been examined by Carnevale et al. by combining classical Molecular Dynamics (MD) simulations and hybrid quantum mechanics/molecular mechanics (QM/MM) methods. MD simulations were performed using methylammonium cation to examine the response of water molecules lying in the pore to an incoming positively charged moiety for the +2 state of the M2 channel (Carnevale et al., 2010). The results disclosed a broad minimum in the free energy landscape in the region below Val27 side chains, and the existence of a second local minimum in the region near Ala34, where the positive charge is stabilized by interactions with water molecules located above the His37 tetrad, and with the local dipoles of the backbone carbonyl groups. QM/MM computations aimed to determine the energetics of proton transport through the set of water molecules located above His37. In this case, the results supported a nearly free diffusion of the excess proton, as the transport of the proton from the water molecules to a neutral His37 occurs through several local minima separated by small barriers. Therefore, these findings suggest that the confining environment in the pore has little impact on the kinetics of proton diffusion, which should resemble proton transport in bulk water.

As a final remark, it is worth noting the computational study of proton conduction reported by Liang et al. (2016), which resorted to mutiscale simulations to follow the proton translocation through the channel considering distinct protonation states (fully deprotonated, +1 and +2) of the channel in models containing the TM region without (M2TM) and with (M2CD) the amphipatic helix, which together define the conductance domain. To this end, the free energy profile for proton migration was determined by combining multiscale reactive molecular dynamics (MS-RMD) and QM/MM MD simulations, which enabled to follow the proton diffusion from the N-terminal and C-terminals ends of the pore as well as (de)protonation of His37 tetrad. The free energy profile determined for the fully neutral and +1 states reveal a high barrier (>15 kcal mol^−1^) for His37 deprotonation to the viral interior, leading to a predicted conductance well below the experimental value, suggesting that the ion channel is inactive at high pH conditions. In the +2 state, however, although the electrostatic repulsion between the incoming proton increases the barrier from viral exterior to His37, deprotonation of His37 is facilitated by the increased electrostatic repulsion between the leaving proton and the His37 tetrad, this process being further assisted by larger exposure to water molecules and increased flexibility of Trp41residues. Thus, the His37 deprotonation barrier is decreased to ∼12 kcal mol^−1^, and the predicted conductance (1.0 and 7.7 for the M2CD and M2TM models, respectively) is in agreement with the measured values (ranging from 0.4 to 4.0; Mould et al., 2000b; Leiding et al., 2010; Sharma et al., 2010).

The whole of these studies illustrate the complexity of gaining a detailed knowledge of the mechanism of proton conduction in spite of the apparent simplicity encoded in the four-helix bundle of the M2 proton channel. The proton conductance determined from electrophysiological assays encompass a variety of molecular events that take place at different spatiotemporal scales, including the formation and breaking of hydrogen-bond complexes implicated in the proton transfer between water molecules and to/from the His37 imidazole-imidazolium ring, the coupling with conformational changes in the side chains of specific residues, and the structural rearrangements observed in the TM helices depending on the protonation state of the His37 tetrad.

### The pKa of Histidines

The mechanistic models described above emphasize the relevance of the microscopic pKa of the His37 tetrad (Table 1) in assisting the proton flow through the inner pore of the M2 channel, which in turn would be associated with the pH-dependent equilibrium between open and closed states of the channel.

**TABLE 1:** Estimated pKa values determined for the His37 tetrad in different constructs of the M2 channel.

Construct	His37 pKas	Lipid environment^a^	References
(22–46)	8.2, 8.2, 6.3, <5.0	DMPC, DMPG	Hu et al. (2006)
(22–46)	7.6, 6.8, 4.9, 4.2	DPPC, DPPE, SM, Chol	Hu et al. (2012)
(21–97)	7.1, 5.4	POPC, POPE, SM, Chol	Liao et al. (2015)
(18–60)	7.6, 4.5	DPhPC	Colvin et al. (2014)
Full length	6.3, 6.3, 5.5	DOPC/DOPE	Miao et al. (2015)

a DMPC: 1,2-dimyristoyl-*sn*-3-phosphocholine; DMPG: 1,2-dimyristoyl-*sn*-glycero-3-phosphoglycerol; DPPC: 1,2-dipalmitoyl-*sn*-glycero-3-phosphocholine; DPPE: 1,2-dipalmitoyl-*sn*-glycero-3-phosphoethanolamine; SM: egg sphingomyelin; POPC: 1-palmitoyl-2-oleoyl-*sn*-glycero-3-phophocholine; POPE: 1-palmitoyl-2-oleoyl-*sn*-glycero-3-phosphoethanolamine; DPhPC: 1,2-diphytanoyl-*sn*-glycerol-3-phosphocholine; DOPC: dioleoylphosphatidylcholine; DOPE: dioleoylphosphatidylethanolamine.

Using ultraviolet Resonance Raman spectroscopy and the transmembrane domain of the M2 proton channel (residues 22–46), Okada et al. reported that the proton channel is activated by low pH with a transition midpoint at pH 5.7 (Okada et al., 2001). Using the same construct, studies by Hu et al. measured pKa values of <5.0, 6.3, 8.2, and 8.2, leading to a +2 protonation state at neutral pH (Hu et al., 2006). Recent studies by Hu et al. supported pKa values of 4.0, 4.2, 6.8, and 7.6, which would support a +3 state at neutral pH, revealing the difficulty of elucidating the protonation preferences of the His37 tetrad and the influence of the experimental conditions used in these assays (Hu et al., 2012). On the other hand, NMR studies performed for a larger construct consisting of residues 21–97, which include the transmembrane helix but also the full cytoplasmic domain, led to pKa values of 7.1 and 5.4 for the second and third protonation (Liao et al., 2015). Likewise, a construct formed by residues 18–60, which encompass both the TM helix and the amphipathic helix that interacts with the interfacial region of the lipid bilayer, yielded estimated pKa values of 7.6 and 4.5 for the second and third protonations (Colvin et al., 2014), whereas pKas of 6.3 and 5.5 for the second and third protonations were determined by Miao et al. for the full-length protein (Miao et al., 2015). Even though the first two pKas are lower than the values estimated for shorter constructs, the authors indicate that at pH 6.2, where the channel should become activated, the M2 channel should be primarily in the +2 state. Moreover, the authors point out that there is little opportunity for a singly charged His37 tetrad.

The differences found in the pKas reported in Table 1 can be ascribed to the usage of different constructs, membrane-mimetic lipid environments, and conformational heterogeneity of the tetrameric helical bundle. In spite of these differences, there is consensus that the first two pKas appear to be above the endosomal pH that activates the M2 channel (pH ∼ 6) (Mould et al., 2000b), suggesting that the physiologically active state for the early activation of the M2 channel corresponds to the protonation state of +2 for the His tetrad.

Computational studies have also been performed with the aim to estimate the pKa of the His37 tetrad, while providing atomistic details of the pH-dependent protonation process. In this regard, Dong et al. performed QM/MM calculations to characterize the structural features of the +3 state and its subsequent deprotonation to the +2 species (Dong et al., 2013; Dong et al., 2014). Calculations were performed considering both a 4-fold symmetric arrangement of the His37 residues (histidine-box) and a 2-fold symmetric configuration (i.e., the dimer-of-dimers arrangement). The results showed that in the triply protonated state the two alternative models (histidine-box and dimer-of-dimers arrangements) converge to a single deprotonation mechanism, and exhibit similar free energy profiles with a barrier height of ∼6.5 kcal/mol to release a proton, supporting deprotonation as a mechanism for proton conduction.

Chen et al. have used constant pH replica-exchange MD simulations to determine the pKa values using a simulation model consisting of the TM helix (Chen et al., 2016). In this study, the simulated system consisted of the TM helix embedded in an explicit DMPC lipid bilayer and surrounded by an explicit solvent model (CHARM22 force field). However, the forces on titration coordinates were determined using the Generalized Born model in conjunction with an implicit membrane. Furthermore, a high-dielectric cylinder that encompass the channel was used to account for the continuous water wire in the pore. Finally, 12 replicas were used in simulations, covering a pH range of 3.5–9.0. The pKa values were estimated to be 8.3, 7.1, 6.2, and 5.7, which are in general close to the first three pKa’s obtained in previous ssNMR studies (see Table 1). The largest deviation is limited to the pKa of the last protonation, likely reflecting limitations arising from the Generalized Born model employed in the hybrid-solvent CpHMD, and to the conformational sensitivity of helices related to differences in the lipid environment used in simulations and experiments.

More recently, Torabifard *et al.* have used CpHMD simulations performed using explicit solvent and the multisite λ-dynamics to estimate the pKa values of the His37 tetrad (Torabifard et al., 2020). They used two models consisting of the TM helix alone (M2TM) and another one that combines the TM region with the amphipatic helix at the interface of the cytoplasmic domain (M2CD). These models were inserted in bilayers composed of a 4:1 ratio of DOPC:DOPE and solvated by water with an ionic force of 0.15 M (CHARMM36 force field). CpHMD simulations were performed using the multisite λ-dynamics approach (Knight and Brooks, 2011). This computational strategy yielded pKa values of 12.5, 10.5, 7.0, and 5.4 for the M2CD model, which are within the range of uncertainty for measured pKa values, especially regarding the first and second pKa values (larger deviations from the experimental data, however, were observed for the M2TM model).

Although the deviations observed between predicted and experimental pKa values may be likely ascribed to inaccuracies in the biomolecular force field, the approximated nature of the physical models used in calculations, incomplete convergence of the CpHMD simulations, and differences in the nature of lipid environments used in simulations and experiments, the results are encouraging and should provide a basis for gaining insight into the molecular factors that underlie the proton conduction in the M2 channel.

### Other Factors That Influence the Proton Conduction of the M2 Channel

Besides the protonation state of the imidazole rings of the His37 tetrad, the efficiency of the M2 channel to perform the proton conduction may be influenced by a number of factors related to both the global structure of the ion channel and to the role of key residues other than His37 and Trp41. In this regard, Asp44 (see Figure 2) may contribute to modulate the proton conduction mechanism, as this residue forms direct or water-mediated hydrogen bonds with the indole moiety of Trp41 in the closed state (Acharya et al., 2010). In fact, Asp44 mutants tend to increase the population of the open state, as there is a loss of a stabilizing interaction of the closed state (Ma et al., 2013).

The influence exerted by the N-terminal ectodomain has been examined by Hong and coworkers (Kwon and Hong, 2016). The ectodomain is highly dynamic, although the motional flexibility is reduced for residues closer to the TM domain, possibly reflecting a tethering effect of the TM helix and the influence of lipid headgroups on the membrane surface. Furthermore, the electrostatic repulsion experienced by acidic residues (i.e., the ectodomain contains four acidic residues and only two cationic residues) appear to promote the adoption of TM conformations that would favor the binding of drugs in the inner pore (see below) even in the absence of the drug. This may be a factor that justifies the lower inhibitory concentration of full-length M2 compared to that of the ectodomain-truncated M2.

In this context, the potential role of Asp21 (see Figure 2) has also been recently highlighted by Jeong and Dyer (2017). Thus, by using a laser-induced pH jump coupled with time-resolved Trp fluorescence spectroscopy, protonation of His37 was estimated to occur in an unusually fast process, as reflected in a protonation rate of 1.6 ± 0.4 × 10^10^ M^−1^ s^−1^, suggesting that Asp21 at the end of the ectodomain and Asp24 at the beginning of the TM helix may act synergistically as proton-collecting antenna residues. The electrostatic field created by these residues would thus create a proton-capturing funnel at the entrance of the ion channel, enhancing proton harvesting from the surrounding aqueous phase. Protons would then be captured by hydrogen-bonded wires of water molecules within the pore, and the hydronium cation could be stabilized by interactions with pore-lining carbonyl groups as well as through bridging water molecules (Thomaston et al., 2015). Furthermore, they concluded that protonation of the His37 tetrad promotes opening of the C-terminal region, enhancing the solvent-exposure of Trp41, with a rate of (4 ± 2) × 10^3^ s^−1^. The temporal decoupling between His37 protonation and this conformational change suggests that probably each proton transport cycle does not require a further conformational change after M2 activation.

Additional studies have examined the effect of the cytoplasmic tail on the conformational properties of the TM domain (Chizhmakov et al., 2003). The results obtained for the cytoplasmic-containing M2 channel reveal that even at neutral pH cationic histidines are present in the interior of the pore, which is in contrast to the results obtained for the TM peptide alone. This effect might be attributed to the acidic character of the cytoplasmic domain, which could facilitate opening of the TM pore at the His37 constriction. Furthermore, the presence of the cytoplasmic domain favors the adoption of a more helical conformation in the His37 backbone, suggesting an ordering effect on the four-helix bundle. Overall, these results provide a basis for the higher proton conductance of full-length M2 channel compared to the TM peptide.

**FIGURE 6:**
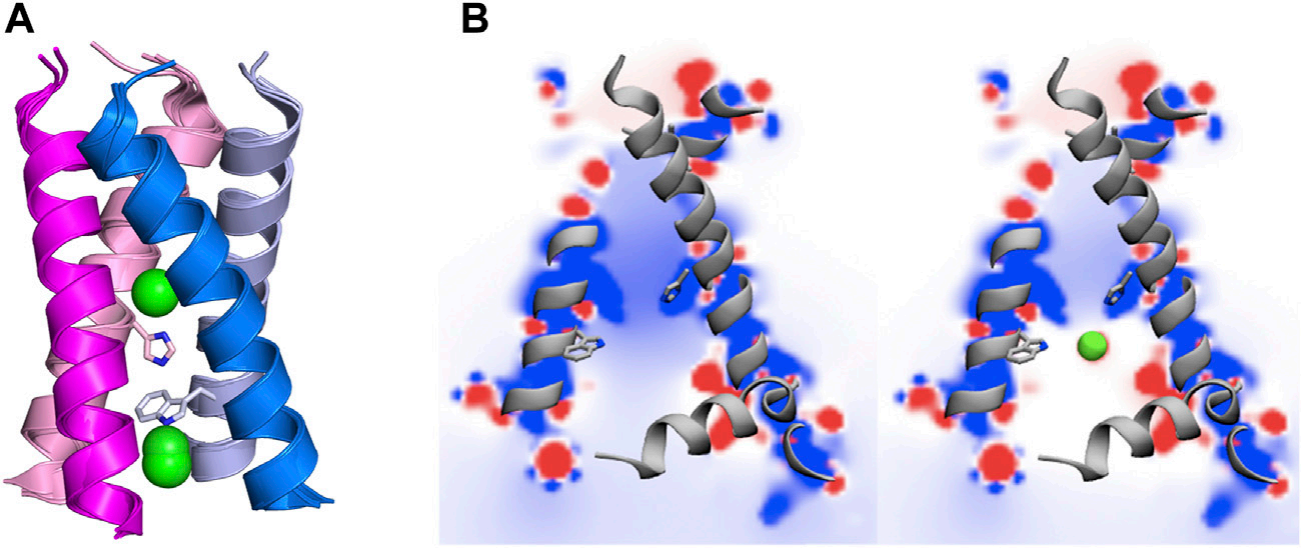
**(A)** Representation of chloride anions (green spheres) in selected X-ray crystallographic structures of the transmembrane region of the M2 proton channel (adapted from PDB entries 5C02, 6BMZ, 6NV1, 6US8 and 6US9). **(B)** Representation of the electrostatic potential (kcal mol^−1^) in a plane passing through the interior of the M2 channel in (left) absence and (right) presence of chloride anions. Isocontours range from +12 (blue) to −12 kcal/mol (red). His37 and Trp41 residues are shown as sticks. Adapted with permission from (Llabrés et al., 2016) (Copyright 2016 American Chemical Society). For the sake of clarify one of the helices is not shown.

Finally, the stability of the protonated states of the His37 tetrad may be affected by the presence of chloride anions in the interior of the pore. Inspection of the X-ray crystallographic data available for the M2 proton channel in the Protein Data Bank (Berman et al., 2000; Burley et al., 2021) reveals the occurrence of a subset of structures where chloride anions are found in the interior of the pore (this subset is collected in Table 2) (Thomaston and DeGrado, 2016; Thomaston et al., 2018; Thomaston et al., 2020; Thomaston et al., 2021). In four out of the five cases, the chloride anion occupies a well-defined position close to the plane formed by the Trp41 residues. Indeed, the chloride anion is stabilized by hydrogen-bond interaction with the indole NH groups, as well as by electrostatic interactions with the positive charge of Arg45 residues, though this latter effect is counterbalanced by the repulsion with the negative charge of Asp44 (Figure 6A). More strikingly, a chloride anion was observed along the four-fold axis in the plane defined by Gly34 residues in the X-ray structure of the S31N mutated variant (PDB entry 5C02).

**TABLE 2:** X-ray structures containing chloride anions at the C-terminus of the M2 proton channel.

PDB ID	Resolution (Å)	Construct (mutant)	Experimental conditions	References
5C02	1.59	22–46 (S31N)	pH: 8.0 T: 283/100 K^a^	Thomaston and DeGrado, (2016)
			LCP (OG)^b^	
6BMZ	2.63	22–46	pH: 7.0 T: 293/100 K	Thomaston et al. (2018)
			LCP (MNG-3-C8)^b^	
6NV1	2.50	22–46 (V27A)	pH: 7.5 T: 293/100 K	Thomaston et al. (2020)
			LCP (MNG-3-C8)	
6US8	1.70	22–46	pH: 7.5 T: 293/100 K	Thomaston et al. (2021)
			LCP (MNG-3-C8)	
6US9	2.00	22–46	pH: 8.5 T: 293/100 K	Thomaston et al. (2021)
			LCP (MNG-3-C8)	

^a^Temperature of crystallization/temperature of data collection (values in Kelvin).

^b^Obtained consideringa lipidic cubic phase (LPC) formed by monoolein and octyl glucopyranoside (OG) or maltose neopentyl glycol analogue (MNG-3-C8).

MD simulations have also revealed the presence of chloride anions in the interior of the channel pore, though the presence of chloride anions depends on the protonation state of the M2 proton channel. Thus, Mustafa et al. reported the occurrence of chloride anions at the level of the Trp41 tetrad for the +2 and +3 states in short simulations performed for the 22–46 construct (Mustafa et al., 2009). Wei and Pohorille also indicated that chloride anions penetrate the pore in all charged states of the M2 channel, but not for the fully unprotonated form (Wei and Pohorille, 2013). The position of the chloride anions was also affected by the charge state, as they were located in the space defined by the His37 and Trp41 planes for the diprotonated form of the channel. Gleed and Busath (2015), Gkeka et al. (2013) and Llabrés et al. (2016) also reported the presence of chloride anions close to the protonated His37 residues.

The presence of chloride anions in the pore may contribute to modulate the structural stability of the helical bundle, as suggested by Wei and Pohorille (2013). The MD simulations performed in presence of chloride ions revealed that they reside near Trp41, but their presence in this location of the pore depends on the charged state of the channel. Thus, anions do penetrate the pore, but to different degree, in all charged states, and they were absent only in the unprotonated form of M2 channel. Furthermore, simulations performed in the presence of phosphate anions confirmed their presence in the pore, suggesting that the size and specific chemical nature of counterions are not essential for the structural stabilization of the channel. Remarkably, additional simulations carried out in the presence of a uniform electrostatic field instead of explicit ions revealed a destabilization of the helical bundle in the +3 and +4 states, reflecting the electrostatic stabilization afforded by counterions that would balance the electrostatic repulsion between protonated His37 residues.

Besides the structural stabilization, the presence of anions in the pore has a strong influence on the nature of the electrostatic potential in the interior of the channel, which is highly positive in the absence of anions, but only slightly positive when chloride anions are present near the His37 sites, according to the MD simulations reported for the channel in the +2 state (Figure 6B) (Llabrés et al., 2016). Keeping in mind the functional relevance of electric fields in enzyme catalysis as well as in (un)binding of ligands to their macromolecular targets (Tan et al., 1993; Dillon et al., 2006; Fried and Boxer, 2017; Vaissier Welborn and Head-Gordon, 2019), the presence of negatively charged ions in the interior of the pore might be valuable to facilitate the diffusion of the proton from the bulk solvent along the N-terminal side of the luminal pore, and hence the transition from the +2 charged species to the triply protonated state. Furthermore, counterions may also facilitate the binding of Amt and related inhibitors by screening the electrostatic repulsion of the protonated amine of inhibitors with the charged His37 residues in the +2 state.

Finally, it is unclear whether the presence of counterions may have a functional role in assisting the translocation of the proton from the protonated His37 residues to the interior of the virion. At this point, it is worth noting that previous QM/MM calculations have suggested that the presence of chloride anions may increase the barrier for deprotonation (Dong et al., 2014), which would reduce the proton conduction. This effect, however, can be largely dependent upon the precise location of the chloride anions relative to the His37-Trp41 pair. Moreover, the functional impact might also be alleviated if the residence time of the chloride anion is affected by the structural fluctuations of the C-terminus in the triply charged state of the channel due to the larger electrostatic repulsion between the imidazolium rings. In particular, one can speculate that a fast exchange of chloride anions might transiently induce an electric field that would ease the transfer of the proton from the protonated His37 tetrad to the C-terminal side, thus facilitating the restoration of the +2 state and hence enabling the entry of new protons to the interior of the virion. In our view, this is an issue that deserves more attention.

## Inhibition of the M2 Proton Channel

Beyond the definition of the molecular events implicated in proton conduction, a major challenge has been the identification of small molecules that can block the proton flow through the inner pore, and hence be used for therapeutic treatments against flu. Amantadine (Amt) and its ethyl analog, rimantadine (Rmt), were patented in 1961 and 1963, respectively (Du Pont Patent, 1961; Prichard, 1967), but they are no longer recommended for the treatment of flu infection. This obeys to several reasons, such as the limited effectiveness against influenza B virus, unwanted side effects, and the emergence of adamantane-resistant influenza A viral strains, primarily single mutated variants V27A, L26F and S31N. These mutations are found in a specific area facing the interior of the pore, suggesting therefore the location of the binding site in the M2 channel (Grambas et al., 1992; Hoyden, 2006; Deyde et al., 2007). For instance, in contrast to the large and widespread chemical shifts observed upon binding of Rmt to the M2 channel, only minor changes in chemical shifts were detected upon addition of a 4-fold molar excess of Rmt to the S31N mutated channel (Cady et al., 2009; Andreas et al., 2010; Andreas et al., 2012). For our purposes here, the discussion of the molecular determinants implicated in drug binding will be limited to the subset of structures available in the Protein Data Bank that contains drug-like inhibitors (see Tables 3, 4).

**TABLE 3:** X-ray and ss-NMR structures of the complexes formed by the wild type M2 proton channel and mutated variants with Amt and Rmt.

PDB ID	Construct (mutant)	Method	Experimental conditions	References
**Amantadine** 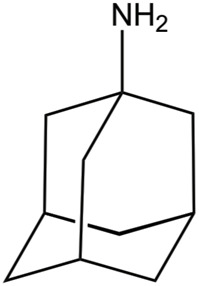				
2KAD	22–46 (L40A)	ss-NMR	pH: 7.5 T: 243 K	Cady et al. (2009)
			DLPC bilayer^a^	
3C9J	22–46 (G34A)	X-ray (3.50 Å)	pH 5.3 T: 298/100 K^b^	Stouffer et al. (2008)
			OG^c^	
2KQT	22–46	ss-NMR	pH 7.5 T: 243 K	Cady et al. (2010)
			DMPC bilayer^d^	
6BKK	22–46	X-ray (2.00 Å)	pH 5.6 T: 293/100 K	Thomaston et al. (2018)
			LCP (MNG-3-C8)^e^	
**Rimantadine** 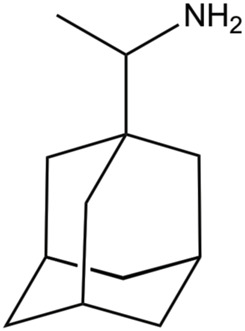				
2RLF (R-Rmt)	18–60	NMR	pH: 7.5 T: 303 K	Schnell and Chou, (2008)
			DHPC^f^	
6BKL (R,S-Rmt)	22–46	X-ray (2.00 Å)	pH 4.5 T: 293/100 K	Thomaston et al. (2018)
			LCP (MNG-3-C8)	
6BOC	22–46	X-ray (2.25 Å)	pH 3.5 T: 293/100 K	Thomaston et al. (2018)
			LCP (MNG-3-C8)	
6US8 (S-Rmt)	22–46	X-ray (1.70 Å)	pH: 7.5 T: 293/100 K	Thomaston et al. (2021)
			LCP (MNG-3-C8)	
6US9 (R-Rmt)	22–46	X-ray (2.00 Å)	pH: 8.5 T: 293/100 K	Thomaston et al. (2021)
			LCP (MNG-3-C8)	

^a^1,2-Dilauroyl-*sn*-glycero-3-phosphatidylcholine.

t ^b^Temperature of crystallization/temperature of data collection in Kelvin.

^c^Octyl-β-D-glucopyranoside.

y ^d^Dimyristoylphosphatidylcholine.

e Structures obtained consideringa lipidic cubic phase (LPC) formed by monoolein and maltose neopentyl glycol analogue (MNG-3-C8).

f Dihexanoylphosphocholine.

**TABLE 4:** X-ray and solution NMR structures of the complexes formed by the wild type M2 proton channel and mutated variants with adamantane-related compounds.

PDB ID	Construct (mutant)	Method	Experimental conditions	References
**(1r,1′S,3′S,5′S,7′S)-spiro [cyclohexane-1,2′-tricyclo [3.3.1.1∼3,7∼]decan]-4-amine** 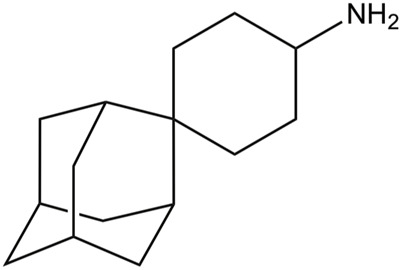				
6BMZ	22–46	X-ray (2.63 Å)	pH: 7.0 T: 293/100 K^a^	Thomaston et al. (2018)
			LCP (MNG-3-C8)^b^	
6NV1	22–46 (V27A)	X-ray (2.50 Å)	pH: 7.5 T: 293/100 K	Thomaston et al. (2020)
			LCP (MNG-3-C8)	
6OUG	21–61 (V27A)	X-ray (3.01 Å)	pH: 8.0 T: 293/100 K	Thomaston et al. (2020)
			LCP (MNG-3-C8)	
**(3S,5S,7S)-N-{[5-(thiophen-2-yl)-1,2-oxazol-3-yl]methyl}tricyclo[3.3.1.1∼3,7∼]decan-1-aminium (M2WJ332)** 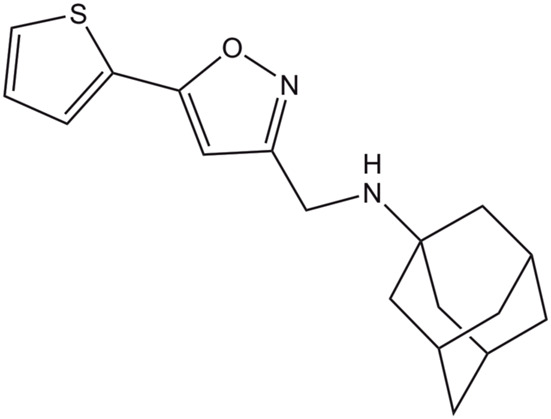				
2LY0	19–49 (S31N)	NMR	pH: 6.8 T: 313 K	Drakopoulos et al. (2018)
			DPC^c^	
**(3S,5S,7S)-N-[(5-bromothiophen-2-yl)methyl]tricyclo[3.3.1.1∼3,7∼]decan-1-aminium** 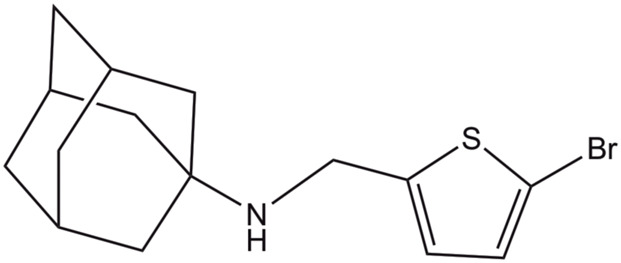				
2MUW	19–49	NMR	pH: 7.5 T: 313 K	Wang et al. (2013)
			DPC	
2MUV	19–49 (S31N)	NMR	pH: 7.5 T: 313 K	Wang et al. (2013)
			DPC	

^a^Temperature of crystallization/temperature of data collection in Kelvin.

c ^b^Structures obtained considering a lipidic cubic phase (LPC) formed by monoolein and maltose neopentyl glycol analogue (MNG-3-C8).

c n-Dodecylphosphocholine.

**FIGURE 7:**
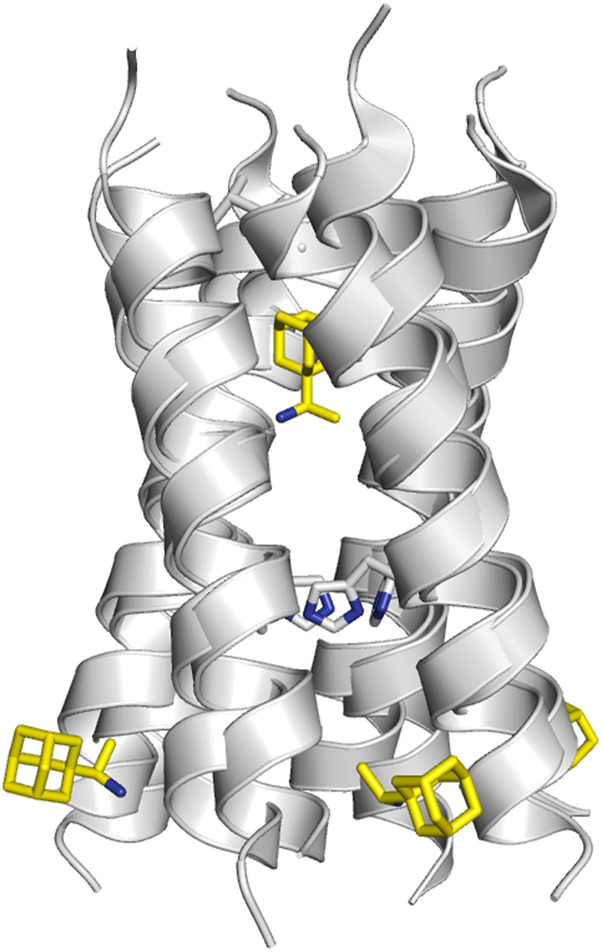
Representation of the binding mode of Rmt (shown as sticks with C-atoms in pink and yellow, respectively) to the TM helical region of the M2 proton channel (residues Val27 and His37 are shown as sticks; obtained from superposition of PDB structures 2RLF and 6BKL).

As noted above, the binding site for M2 inhibitors is located in the interior of the pore, filling a pocket shaped by residues Val27, Ser31, Gly34, and His37. Early neutron difraction (Duff et al., 1994) and computational (Samson and Kerr, 1993) studies already identified this pocket as the binding site that mediates the blockade of proton conduction by Amt. Detailed information about the binding mode of Amt and Rmt has subsequently been gained from structural studies based on X-ray diffraction, solution NMR and ss-NMR (Table 3; Schnell and Chou, 2008; Stouffer et al., 2008; Cady et al., 2009; Cady et al., 2010; Thomaston et al., 2018). The inspection of these structures reveals the existence of two binding sites (Figure 7). In most cases the drug is located in the interior of the pore filling the space located between Val27 and Gly34 (pore-binding model), but in two structures (PDB ID 2RLF and 6US8) the drug is also found outside the channel located in a peripheral site close to Leu43 and Asp44 in the C-terminus of the TM helical region (interface-binding model). The high-affinity site lies in the pore, as revealed from the analysis of REDOR dipolar dephasing between ^13^C-labeled M2TM and perdeuterated Amt in ss-NMR studies performed in a lipid bilayer (Cady et al., 2010). From a computational point of view, free energy calculations also pointed out that binding of Rmt to the pore-binding site is ∼7 kcal/mol more favorable relative to the interface-binding site, leading to more stable drug binding and channel inhibition (Li et al., 2008). The presence of the peripheral binding site can be attributed to the partitioning of the drug in the membrane-like lipidic environment (Duff et al., 1994; Gu et al., 2011). The studies reported by Cady et al. (2010) showed that the highest affinity binding site of amantadine is the N-terminal pore lumen, which is consistent with the known stoichiometry of binding (1:1 drug:channel) and the location of resistant mutations, such as V27A and S31N. However, increasing the drug:channel concentration up to 4:1, the drug contributes ∼7% of the amphiphiles composing the DMPC bilayer, and binding to the low-affinity, peripheral site is observed in NMR studies. Thus, when free amantadine is a major component of the membrane, Amt contacts the C-terminus of the protein, though the affinity for the peripheral site has been estimated to be ∼40-fold lower (see (Wang et al., 2015) for a detailed discussion of biophysical, computational and functional assays that addressed the binding mode of Amt and Rmt).

Inspection of these structures shows that in the pore the drug is oriented along the axis of the channel. Drug binding causes a dehydration of the channel, thus preventing the formation of a water wire from the N-terminus, although the amino group of Amt and Rmt is bound to water molecules that fill the space located between the drug and the His37 tetrad. In the specific case of Rmt, the two enantiomers bind to the pore with only slight differences in the hydration pattern, and exhibit similar values for association and dissociation kinetic rate constants and binding affinities, which was in agreement with the small differences in the relative binding free energy (around 0.3 kcal mol^−1^) determined from free energy calculations (Thomaston et al., 2020). Furthermore, Rmt was found to be more potent than Amt, as noted in a ∼14-fold ratio of their binding affinities to the M2 channel. On the other hand, it has been shown that binding of Amt reduces the pKa of His37 and alters the propensity to form hydrogen-bond interactions in the His37 tetrad (Hu et al., 2007; Cady et al., 2011).

From a computational standpoint, fine details of the binding mode of adamantane blockers have been extensively investigated. Yi et al. paid attention to the changes in conformational states of the M2 channel upon binding of Amt through comparison of the ensembles collected for both apo and bound forms (Yi et al., 2008; Yi et al., 2009). In particular, they observed a reduction in the conformational heterogeneity upon Amt binding, as reflected in a narrower distribution of kink angles around Gly34 in the transmembrane helices compared to the apo species, which agrees with the broadened resonances observed from ssNMR studies of this latter form. In contrast, large-kink angles were not observed in simulations. The occurrence of kinks also allows the access of water molecules that may stabilize the kinked helices via hydrogen bonds with the backbone carbonyl and amide groups around the kink, which in turn may also influence the proton conduction.

On the other hand, efforts have been conducted to predict the differences in binding affinity for adamantane inhibitors using free energy calculations, including implicit solvent/implicit membrane molecular mechanics Poisson-Boltzmann surface area (MM-PBSA) approach (Homeyer et al., 2016) and free energy perturbation techniques (Gkeka et al., 2013; Ioannidis et al., 2016). Gratifyingly, alchemical free energy calculations performed for a set of 10 adamantane ligands reproduced satisfactorily the binding potency determined experimentally using isothermal titration calorimetry (ITC) against the M2 channel at high pH (pH = 8), especially keeping in mind that they cover a binding affinity range of only ∼2 kcal mol^−1^. Nevertheless, it is worth noting that the agreement between computational and experimental data was affected by the nature of the lipids used to model the membrane. In particular, a higher correlation between experimental and computed relative binding free energies was obtained when calculations were performed for the tetramer in a 1,2-dimyristoyl-*sn*-glycero-3-phosphocholine bilayer, possibly due to a larger mimetic resemblance with the dodecylphosphocholine micelles used for ITC measurements.

**FIGURE 8:**
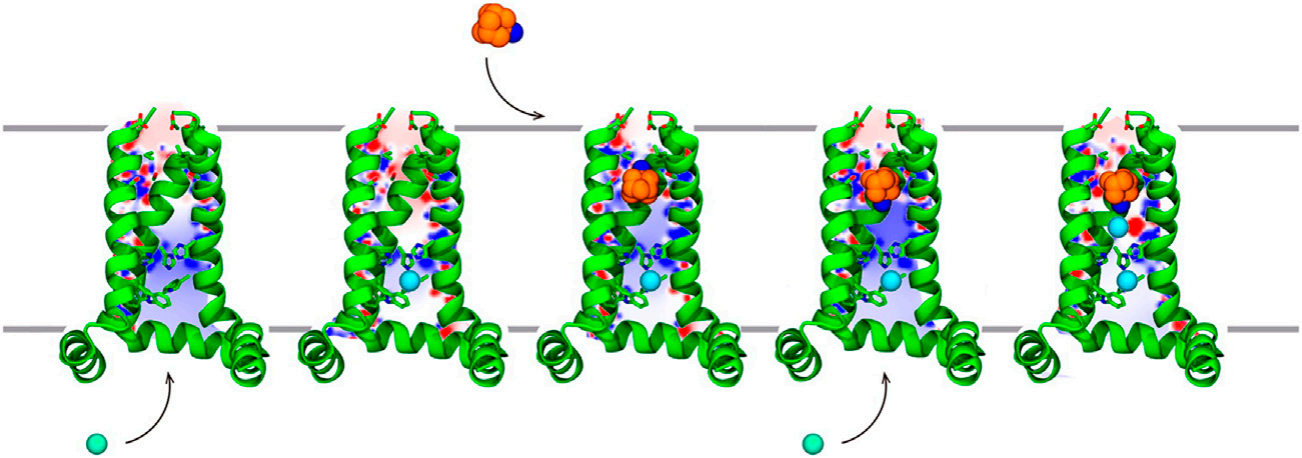
Representation of the stepwise mechanism for Amt (carbon/nitrogen atoms shown as orange/blue-colored spheres.) binding to the M2 proton channel. (i) Amt is trapped by electrostatic interactions with Asp24. (ii) Thermal fluctuations of the helices facilitate crossing through the Val27 filter, and Amt fills the channel lumen in the *up* binding mode (iii) Finally, rotation of Amt leads to the *down* state, which is further stabilized by chloride anions. Chloride anions are shown as green spheres. For the sake of clarity, only three helices of the tetrameric channel are shown as green cartoon embedded in the lipid membrane. Reprinted with permission from (Llabrés et al., 2016) (Copyright 2016 American Chemical Society).

Furthermore, the entire recognition process of Amt to the M2 channel has been explicitly explored using multiple-walkers well-tempered metadynamics calculations (Llabrés et al., 2016). The results suggested that Amt retains some degree of conformational motion in the pore allowing the adoption of two main orientations where the amino group is oriented to the N-terminus and C-terminus (denoted *up* and *down*, respectively). Binding of Amt would follow a sequential mechanism that would involve trapping of the protonated Amt by the negative electrostatic field created by the tetrad of Asp24, followed by the adoption of a transiently populated intermediate with Amt in the *up* state, and finally the reorientation in the interior of the pore to the *down* state, which is the thermodynamically favored orientation (Figure 8). Release of Amt from the *down* state to the channel mouth is destabilized by ∼12 kcal mol^−1^. The agreement with the experimental binding affinity (∼8–9 kcal mol^−1^) is reasonable, since the theoretical estimate omits the free energy contribution due to the release of Amt to the aqueous solvent, and the fact that the apparent *K*_*i*_ might not properly correspond to an equilibrium measurement of the inhibitory constant (Wang et al., 1993; Ma et al., 2009; Rosenberg and Casarotto, 2010). As a particular remark, this study also pointed out that the *down* → *up* transition depends on the presence of chloride anions in the C-terminus of the channel, since the stabilization of the *down* state can be enhanced by ∼6 kcal mol^−1^ due to the modulation of the electrostatic field in the interior of the pore. Finally, the barrier from Amt dissociation was estimated to be ∼19 kcal mol^−1^, which compares a value of 22 kcal mol^−1^ determined from electrophysiological assays (Wang et al., 1993).

**FIGURE 9:**
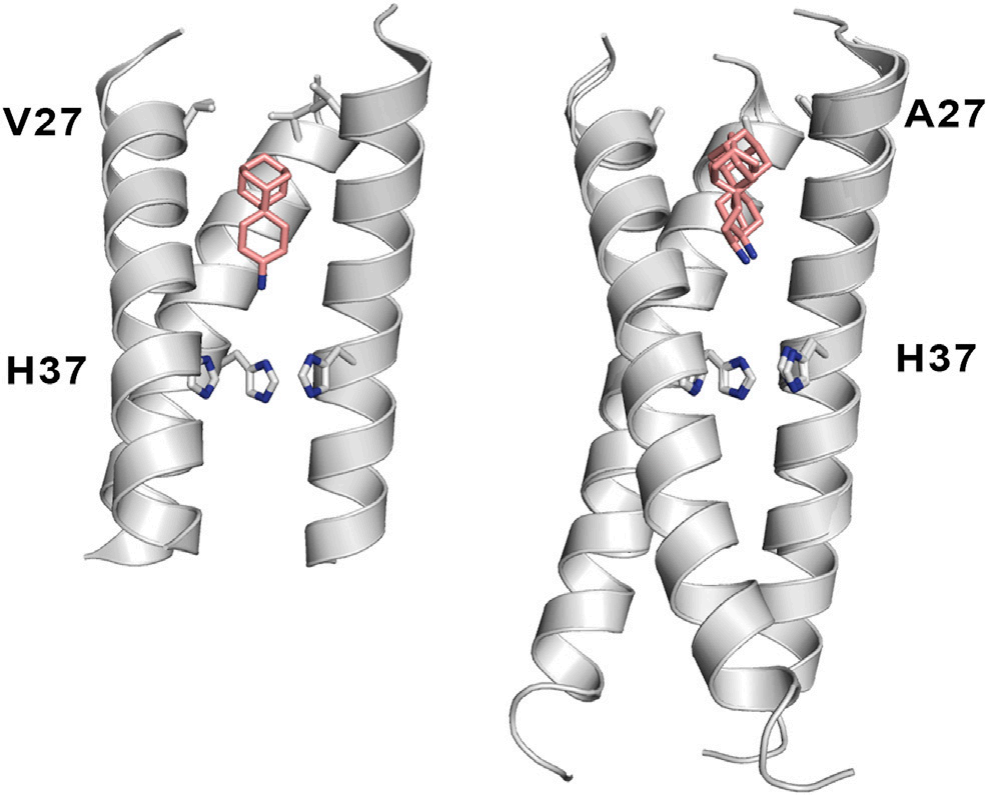
Representation of the binding mode of the spiroadamantane **1** bound to (left) the wild type M2 proton channel (construct 22–46; PDB ID 6BMZ) and (right) the V27A mutant (construct 21–61; PDB ID 6NV1 and 6OUG). The ligand is shown as sticks with C-atoms in pink, and the TM helical region of the M2 proton channel as cartoon (residues Val27, Ala27 and His37 are shown as sticks). For the sake of clarify one of the helices is not shown.

Mutation of Val27 to Ala causes a drastic alteration in the free energy surface for Amt binding, as noted in a stabilization of the *up* state less than 1 kcal mol^−1^, which agrees with the experimentally observed lack of inhibitory potency for the Amt-resistant V27A channel. This effect can be attributed to the increased accesible volume in the inner pore due to the V27A mutation, which encouraged the search of compounds with an expanded hydrophobic cage (Duque et al., 2011; Wang et al., 2011; Rey-Carrizo et al., 2014; Rey-Carrizo et al., 2015; Thomaston et al., 2018). In the case of the spiroadamantane **1** ((1r,1′S,3′S,5′S,7′S)-spiro [cyclohexane-1,2′-tricyclo [3.3.1.1∼3,7∼]decan]-4-amine) shown in Table 4 (Thomaston et al., 2018; Thomaston et al., 2020), comparison of the X-ray structures solved for the wild type and the V27A mutant shows that the compound exhibits a similar arrangement in the ion channel, which is found in the closed conformation (Figure 9) (Thomaston et al., 2020). The ligand exhibits only a slight shift depending on the nature of the residue at position 27. In the V27A complex, the amino group of **1** occupies approximately the same position as the ammonium group of Amt in complex with the wild type channel (PDB entry 6BKK) (Thomaston et al., 2018). This is facilitated by the larger free volume enabled by the presence of Ala27. Nevertheless, in the complex with the wild type channel, the adamantyl group of **1** and Amt overlap, but the ammonium group binds deeper in the pore at the expense of displacing few water molecules. MD simulations performed for the V27A complex with **1** reflected the shift of the ligand toward the N-terminus.

A combination of computational techniques, biophysical studies, functional assays and classical medicinal chemistry approaches has been used also to design potent inhibitors of V27A. This mutation imposes a reduction in the steric constraint found in the wild type channel, suggesting the compounds with a size-expanded hydrophobic cage would be better suited to fit the larger volume of the pocket in the V27A channel (Wang et al., 2011). An example is the spiroadamantane **4**, which has an IC_50_ value of 18.7 μM against the wild type channel and of 0.3 μM against the V27A species. MD simulations showed that the ammonium group occupies the lower aqueous site forming solvent-mediated hydrogen bonds with the His37 residues in both wild type and V27A, but in this latter case the compound was slight shifted toward the N-terminus due to the extra space afforded by Ala27.

**FIGURE 10:**
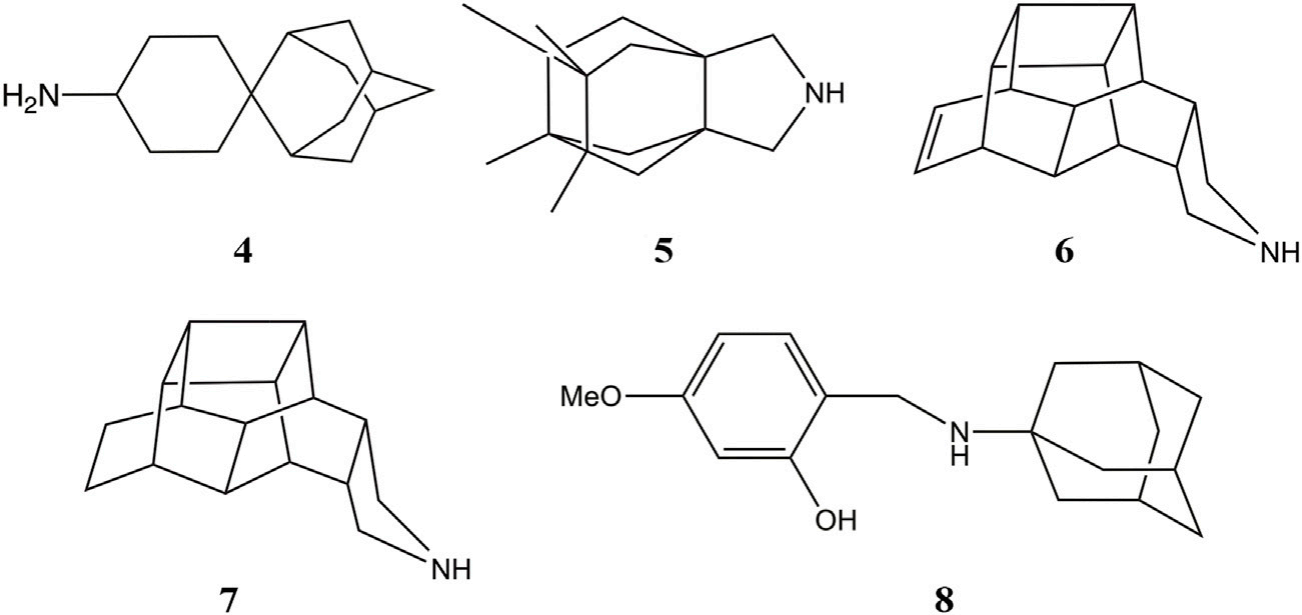
Representation of selected size-expanded analogs of Amt.

Other efforts to develop size-expanded analogs have led to the pyrrolidine derivative **5** reported by Vázquez and coworkers (Figure 10) (Duque et al., 2011; Rey-Carrizo et al., 2014). This compound inhibits the wild type M2 channel with an IC_50_ of 18 μM, while being *ca*. 26-fold more potent against the V27A mutant. MD simulations revealed an orientation consistent with the *down* state of Amt, with the amine nitrogen pointing toward the His37 tetrad with an average tilt angle (i.e., the deviation of the amine nitrogen from the pore axis) of 16°. Nevertheless, in the V27A complex **5** adopted the *down* orientation, but was also found in a *up*-like arrangement, presumably facilitated not only by the larger volume in the pore, but also by the widening of the helices at the location of Ala27, as the cross-diagonal distance between Cα atoms at position 27 was enlarged by 1.1 Å compared to the wild type channel. The effect of including other polycyclic scaffolds as the hydrophobic cage have led to analogs **6** and **7** (Figure 10), which exhibit a micromolar potency against the wild type channel (IC_50_ ∼ 2 μM), but a lower inhibition in the V27A mutant (IC_50_ ∼ 17.2 and 184.6 μM for **7** and **6**, respectively) (Rey-Carrizo et al., 2015), suggesting a limiting effect in the size of the hydrophobic cage even for the V27A channel. At this point, MD simulations suggested that even the center-of-mass of **6** and **7** was located between the planes formed by S31 and A27 tetrads, the protonated amine was pointing to the N-terminus in five out of six MD simulations.

The resistance of the S31N channel, which is the most prevalent in currently circulating influenza viruses, to inhibition by Amt is reflected in the more variable motion and orientation of the ligand in the mutated channel. Thus, whereas Amt occupies a stable position in the wild type channel, it showed larger positional fluctuations in the pore of the mutated channel. This trait is reflected in a decreased energetic stability of the ligand in the proximity to the Asn31 side chains, as reflected in potential of mean force calculations. Finally, this destabilization was accompanied by an increase in the density of water molecules around and above Amt in the S31N channel compared to the wild type species, enabling water molecules to fill laterally the available space in the channel pore (Gleed and Busath, 2015). Furthermore, studies conducted for the binding of Rmt to the wild type M2 channel and the S31N variant have related the resistance with the higher dissociation rate constant determined for the mutated channel (Drakopoulos et al., 2018). In turn, this effect was attributed to the reduction in van der Waals interactions due to the shift of the ligand toward the C-terminus due to due to enhanced repulsive forces of the Asn side chains with the adamantyl ring in the mutated channel, and the concomitant weakening of the stabilizing contacts with Val27.

While these features reveal the challenges for developing inhibitors against the M2 S31N variant, significant progresses have been made in the last years. An example is compound **2** ((3S,5S,7S)-N-{[5-(thiophen-2-yl)-1,2-oxazol-3-yl]methyl}tricyclo [3.3.1.1∼3,7∼]decan-1-aminium), also denoted M2WJ332, Table 4). This compound inhibits the proton conduction in electrophysiological assays of the S31N channel with an IC_50_ of 16 μM, being however much less effective against the wild type ion channel (Rey-Carrizo et al., 2014). Remarkably, the NMR structure reveals that the compound binds the S31N channel in a different orientation compared to Amt and Rmt (Figure 10), since the adamantly ring is located facing the His37 tetrad, whereas the thienyl group is located at the level of the Val27 tetrad. This arrangement is facilitated by the slight expansion (∼1.0 Å) observed at the N-terminus of the TM helices relative to the wild type M2 channel. As noted in the analysis of MD simulations performed for the complex with **2**, this binding mode is reinforced by hydrogen-bonds formed by the protonated amine and the isoxazole ring with three Asn31 residues (Wang et al., 2013).

**FIGURE 11:**
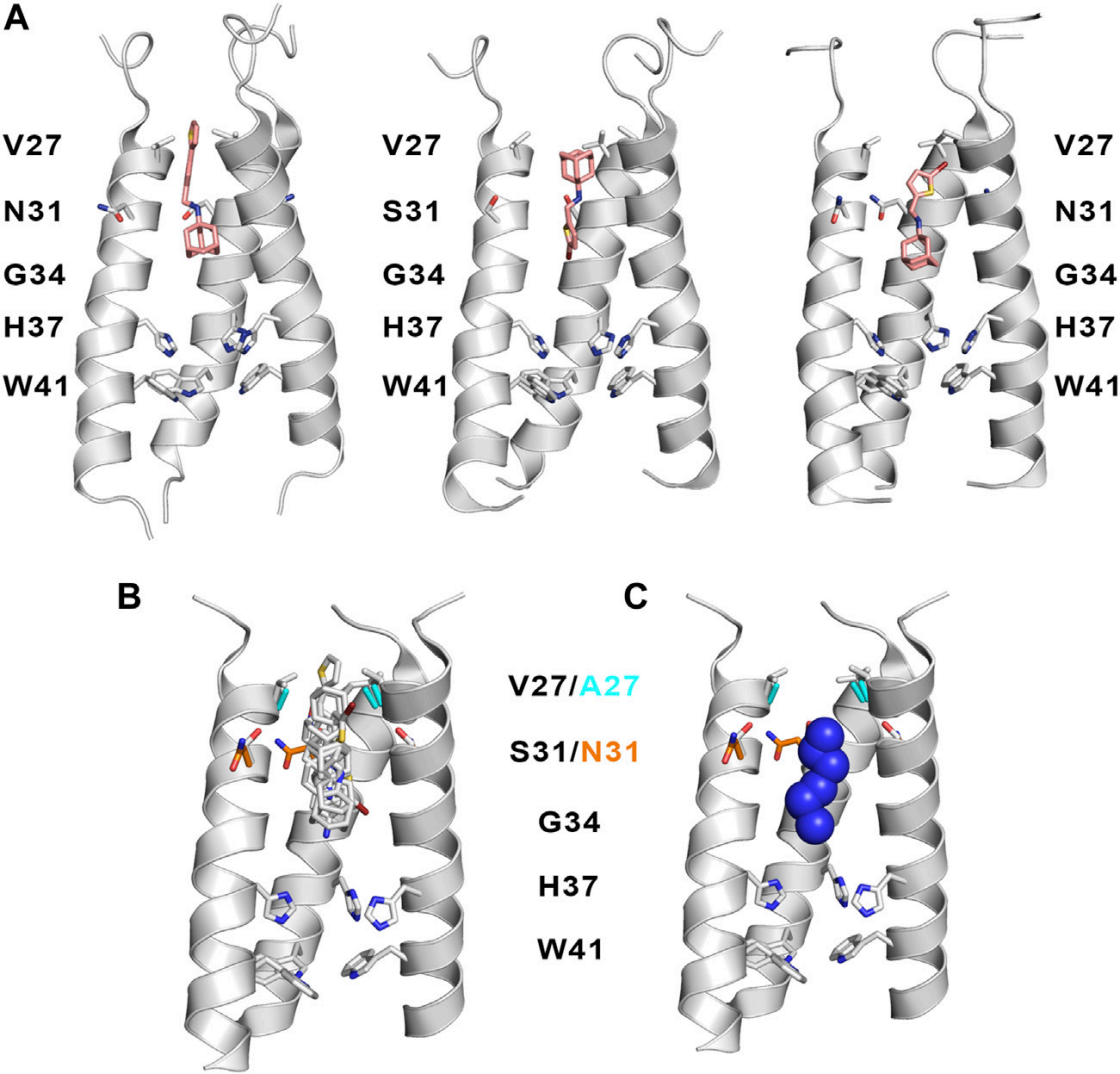
**(A)** Representation of the binding mode of (left) compound **2** bound to the S31N channel (construct 19–49; PDB ID 2LY0), and compound **3** bound to (middle) the wild type M2 proton channel (construct 19–49; PDB ID 2MUW) and (right) the S31N mutant (construct 19–49; PDB ID 2MUV). The ligand is shown as sticks with C-atoms in pink, and the TM helical region of the M2 proton channel as cartoon (residues Val27, Ala27 and His37 are shown as sticks). For the sake of clarify one of the helices is not shown. **(B)** Superposition of selected inhibitors of wild type M2 ion channel and its V27A and S31N variants (taken from PBD ID 2KQT, 6US8, 6BMZ, 6NV1, 2LY0, 2MUW, and 2MUV). **(C)** Representation of the location of the protonated amine nitrogen atom along the channel axis taken from the previous subset of structures. For the sake of simplicity, only three helices of the tetrameric bundle are shown using the backbone skeleton of PDB ID 2KQT as cartoon is shown in plots **(B**,**C)**.

Starting from compound **2**, a successful rational design led to compound **3** ((3s,5s,7s)-N-[(5-bromothiophen-2-yl)methyl]tricyclo [3.3.1.1∼3,7∼]decan-1-aminium; Table 4) (Drakopoulos et al., 2018), which exhibits a similar potency in inhibiting both the wild type and the S31N channel (around 77% inhibition in two-electrode voltage clamp assays, and EC_50_ values of 4.6 and 1.8 μM, respectively). The chemical scaffold of compound **3** combines an adamantane moiety with a bromothiophene unit. However, the most remarkable structural feature is that **3** adopts completely flipped arrangements in the wild type and mutated channels (Figure 11A). Thus, the bromothiophene unit of the ligand faces the His37 tetrad in the wild type channel, but it is pointing toward the N-terminus in the S31N variant. MD simulations confirmed the structural stability of the flipped orientations in the wild type and mutated channels, enabling an interpretation of the structure-activity relationships (Wu et al., 2014). Inspection of the NMR structures (2MUW and 2MUV) does not support the involvement of halogen bonding, as this noncovalent interaction requires specific geometric features between the halogen atom and an electron-rich atom that are not fulfilled in these structures (see Kolár and Hobza, 2016 for details of halogen bonding). Furthermore, MD simulations revealed the formation of weak interactions between the bromine atom and water molecules, supplemented with transient, nonspecific contacts with the imidazole ring of His37 and the carbonyl group of Gly34 in the wild type channel, and with the Val27 side chains in the S31N channel (Wu et al., 2014).

It is also interesting to highlight the synthesis of aminoadamantane-CH_2_-aryl derivatives (**8** in Figure 10) designed as sensitive probes for blockage of the wild type and S31N ion channels (Tzitzoglaki et al., 2020). These compounds retain the aminoadamantane unit present in **2**, but the isoxazole ring has been replaced by a substituted phenyl ring. MD simulations have shown that compound **8** exhibits a stable binding in the S31N mutated channel. The binding mode locates the adamantyl group between Val27 and Gly34, and the phenyl unit fills the space between the side chains of Val27 residues. The results obtained from electrophysiological assays in oocytes evidence that blockage of the proton conduction in the wild type channel is relatively insensitive to chemical changes in the scaffold of **8**, such as the insertion of a methylene between the adamantane and amino units or the replacement of adamantane by diamantane or triamantane. However, these changes have a drastic effect on the current blockage of the S31N channel, leading to lower association and higher dissociation rate constants relative to the parent compound **8**. These results point out the larger sensitivity of the S31N ion channel to the chemical features of inhibitors upon binding to the pore.

Superposition of the compounds shown in Tables 3, 4 is useful to exemplify the ability of the inhibitors to exploit different sites and binding modes along the interior of the pore in order to prevent proton transport in the wild type M2 channel and the V27A and S31N mutated species. (Figures 11B,C). This feature has been highlighted in previous studies that combined the structural analysis of bound channels with metadynamics calculations (Wang et al., 2013; Gianti et al., 2015), which pointed out the existence of distint locations that can be occupied by the protonated amine along the axis of the channel. These studies also remarked the role played by waters molecules inside the pore, as they contribute to stabilize the inhibitor by facilitating hydrogen-bond bridges with carbonyl groups of the transmembrane helices as well as with the His37 tetrad. From a practical perspective, the presence of multiple interaction sites for the protonated amine and the structure of water clusters that stabilize the inhibitor can be useful hallmarks for guiding the design of new drugs. In this regard, recent studies have indicated that the ammonium group of these inhibitors can act as a hydronium mimic upon binding to the channel pore, suggesting that the adamantane-like compounds function as mechanism-based inhibitors as long as they exploit structural features of the channel implicated in proton transport (Watkins et al., 2020).

As a final remark, it is worth noting a very recent study by Kolokouris and coworkers where a systematic analysis of setup conditions and methodological details, covering from docking calculations to MD simulations, is presented for the M2 channel (Kolocouris et al., 2021). This study examines distinctive traits, such as the usage of M2 (22–46) and M2 (22–62) constructs, lipid content in the bilayer (1,2-dimyristoyl-sn-glycero-3-phosphocholine,1-palmitoyl-2-oleoyl-*sn*-glycero-3-phosphocholine, and 1-palmitoyl-2-oleoyl-sn-glycero-3-phosphoethanol-amine), and the effect of cholesterol, paying particular attention to the location of channel blockers and water structure, the presence of chloride anions, structural properties of the helical bundle, and the binding site of cholesterol. While this study deserves practical interest for the choice of parameters in simulations of the M2 proton channel (and related viroporins), we limit ourselves to highlight three findings. First, the results obtained for M2 complexes with Amt at pH 8 revealed that chloride anions approached Arg45 and occupied a position located between Trp41 and Arg45 (at a distance close to 15.5–17.5 Å from the Amt nitrogen atom) presence of chloride anions stabilized in the interior of the pore. The anions approached in the C-terminus. The anions approached Arg45. Second, the simulations with CHARMM36 preserved the structure of the amphipatic helices, providing a correct description for the wedge shape geometry that might be required to modulate the saddle-shape curvatuve of the membrane for the release of virions (Wang and Hong, 2015b; Martyna et al., 2017). Finally, the ability of cholesterol to interact with the tetrameric channel, forming van der Waals interactions with Ile39, Leu40, Leu43, Leu46, Phe47, Ile51 and Phe54, and a hydrogen bond with Ser50, which is in agreement with previous experimental evidence (Ekanayake et al., 2016; Elkins et al., 2017; Elkins et al., 2018), paving the way to future studies about the role of cholesterol in mediating the M2 protein function.

## Conclusion

Resistance to current pharmacological treatments is a severe health challenge, as illustrated by the emergence of viral strains resistant to drugs interfering with the M2 proton channel, such as Amt and Rmt, but also to antiviral agents targeting neuraminidase, such as oseltamivir, as noticed in a resistant strain associated to the His275Tyr mutation in this protein (Meijer et al., 2009; Ginex and Luque, 2021). Currently circulating seasonal H1N1 and H3N2 strains are now resistant to adamantanes, and oseltamivir is no longer effective against the pandemic H1N1 subtypes (Hussain et al., 2017).

Finding new active antiviral compounds is an urgent need, which may be explored resorting to the application of a combinatorial therapy that should potentiate the effect of two or three drugs endowed with distinct mechanisms of action, hopefully being less susceptible to inactivating mutations (Dunning et al., 2014). A long-term strategy is the identification and characterization of the molecular mechanisms associated to the activity of proteins relevant for the life cycle of the virus, as this knowledge may disclose novel strategies for the design of antiviral compounds.

This approach is illustrated by the research efforts invested in the M2 proton channel. In spite of the apparent simplicity posed by the membrane-embedded four-helix bundle, the flow of protons reflects a complex synergy between different molecular events, involving the acidity of the His37 residues, the structural coupling between His37 and the gating Trp41 residue, the structure of the hydrating water molecules in the pore, the pH-dependent conformational arrangement of the TM helices, the electrostatic funnel created by acidic residues in the N-terminus of the channel, and the influence of the amphiphilic helix and cytoplasmic tail on the flexibility of the TM domain, in addition to the potential influence exerted by other factors such as the nature of the lipid environment in the viral capside.

Although a full understanding of the interplay between these factors, which encompass a diverse range of spatial and time processes, has not yet been achieved, the progress consolidated in the last decades through the combined use of structural, biophysical, physiological and computational techniques is impressive, and the successful development of compound **3** is encouraging. This scientific background defines the framework to address novel questions about the mechanisms of drug resistance and the guidance to novel antiviral and treatment approaches, such as the potential dependence between drug resistance and the nature of the viral strain or the infected cell type, which have been the subject of recent studies (Musharrafieh et al., 2019; Musharrafieh et al., 2020). Hopefully, the outcome will be valuable to enrich our current anti-influenza therapeutic arsenal in the form of effective antivirals less susceptible to drug resistance.
